# Triciribine Engages ZFP36L1 and HuR to Stabilize LDLR mRNA

**DOI:** 10.3390/molecules25194505

**Published:** 2020-10-01

**Authors:** Hilde Sundvold

**Affiliations:** Unit for Cardiac and Cardiovascular Genetics, Department of Medical Genetics, Oslo University Hospital, 4953 Oslo, Norway; hisund@ous-hf.no

**Keywords:** AKT, LDLR, TCN, mRNA-stability

## Abstract

An increased understanding of low-density lipoprotein receptor (LDLR) and its regulation may facilitate drug development for the treatment of hypercholesterolemia. Triciribine (TCN), which is a highly selective AKT inhibitor, increases the stability of LDLR mRNA downstream of extracellular signal-regulated kinase (ERK) in human hepatoma cells (HepG2). Here, a candidate approach was used in order to determine whether the RNA-binding proteins (RBPs) ZFP36 ring finger protein like 1 (ZFP36L1) and Hu antigen R (HuR) play a role in TCN-mediated stabilization of LDLR mRNA. The depletion of HuR led to a reduction of LDLR mRNA stability, an event that was more pronounced in TCN-treated cells. TCN was found to induce the translocation of nuclear HuR to cytoplasm in an ERK-dependent manner. ZFP36L1 depletion increased the stability of LDLR mRNA consistent with its destabilizing role. However, in contrast to HuR, TCN had no effect on LDLR mRNA turnover in ZFP36L1-depleted cells. TCN induced the phosphorylation of ZFP36L1 in an ERK/RSK-dependent manner and promoted its dissociation from the CCR4-NOT complex. In sum, these data suggest that TCN utilizes ERK signaling to increase the activity of HuR and inhibit ZFP36L1 to stabilize LDLR mRNA in HepG2 cells.

## 1. Introduction

Low-density lipoprotein receptor (LDLR) mediates the cellular uptake of plasma low-density lipoprotein cholesterol (LDL-C), the elevated levels of which are associated with an increased risk of cardiovascular disease. The number of LDLR that is expressed on the surface of hepatocytes is the major determinant of the circulating levels of LDL-C [1,2]. The LDLR levels are regulated by an array of mechanisms that operate at the transcriptional, posttranscriptional, and posttranslation levels. The transcription of LDLR is controlled by the transcription factors, called sterol regulatory element-binding proteins (SREBPs), whose activities are regulated by the intracellular levels of cholesterol [1,2]. At the posttranslational level, LDLR protein stability is subject to regulation by both proprotein convertase subtilisin/kexin 9 (PCSK9) and inducible degrader of LDLR (IDOL) [3,4]. In addition to the regulation of gene transcription, LDLR levels are dynamically regulated by the modulation of its mRNA turnover in response to extracellular stimuli [5,6]. LDLR mRNA has a half-life of approximately 1–2 h in HepG2 cells and it becomes stabilized two- to three-fold in response to extracellular stimuli, including phorbol 12-myristate 13-acetate (PMA) [7], bile acids [8,9], berberine (BBR) [5,10,11], and triciribine (TCN) [12]. Whereas TCN inhibits the serine/threonine protein kinase (AKT) signaling pathway and PMA activates protein kinase C (PKC), both compounds modulate signaling pathways related to cell growth. Therefore, several clinical studies have been performed, and are in progress, to evaluate TCN and PMA as pharmacological compounds in the treatment of cancer, amongst other diseases (ClinicalTrials.gov).

The regulation of LDLR mRNA decay is controlled through its 3′UTR, which consists of four adenylate uridylate-rich element (AREs) functioning in mRNA decay by binding a network of RNA-binding proteins (RBPs) [13,14]. Among these, Hu antigen R (HuR) is one of the few RBPs identified that confers stability to LDLR mRNA [9,13,14] whereas ZFP36 ring finger protein like-1 and -2 proteins (ZFP36L1 and ZFP36L2) and KH-type splicing regulatory protein (KSRP) accelerate the turnover of LDLR mRNA [13,15]. Other RBPs, such as D and I members of the heterogeneous nuclear ribonucleoprotein family (hnRNP D and hnRNPI), either stabilize or destabilize LDLR mRNA, depending on their isoform expression profiles and cellular context [16,17,18,19].

HuR is a ubiquitously expressed member of the embryonic lethal abnormal vision family of RBPs [20]. HuR is predominantly localized to the nucleus where it carries out various functions, such as the regulation of pre-mRNA processing [21,22]. However, when translocated into the cytoplasm, HuR exerts a stabilizing effect on its mRNA targets [22,23,24]. One such target is LDLR mRNA [13], which is stabilized in an HuR-dependent manner in response to farnesoid X receptor activation and the AMPK activator, AICAR [9,14]. Interestingly, the stabilizing effect of HuR on LDLR mRNA in response to AICAR appears to require the activity of ERK [14].

ZFP36L1 and ZFP36L2 belong to the TPA-inducible sequence 11 (TIS11) protein families that, upon binding to AREs in the 3′ UTRs of their target mRNAs, recruit the CCR4-NOT complex to the adjoining polyA tail, thus promoting the deadenylation of mRNA and ultimately its degradation [15]. Phosphorylation plays a critical role in regulation of the activity of TIS11 family members [25]. For instance, phosphorylation of ZFP36 by MK2, a kinase downstream of p38 MAPK, has been found to diminish its mRNA-destabilizing activity [26]. Similarly, the phosphorylation of ZFP36L1 and ZFP36L2 by p90 ribosomal S6 kinase 1 (RSK1), a downstream effector of ERK signaling, has been shown to induce their disengagement from CCR4-NOT complex and the loss of its de-stabilizing function, resulting in the stabilization of LDLR mRNA [15].

Recently, it was reported that TCN increases LDLR levels by preventing the degradation of LDLR mRNA in an ERK activity-dependent manner [12]. This work aimed to elucidate the mechanism that underlies this property of TCN. To do so, a candidate protein approach was adopted, and attention was directed towards trans-acting factors that, in addition to lying downstream of ERK, have been demonstrated to alter the stability of LDLR mRNA. In this report, it was shown that both HuR and ZFP36L1 contribute to the stabilizing effect of TCN on LDLR mRNA.

## 2. Results

### 2.1. HuR and ZFP36L1 Modulate Endogenous LDLR Expression and mRNA Stability in HepG2 Cells

**Figure 1:**
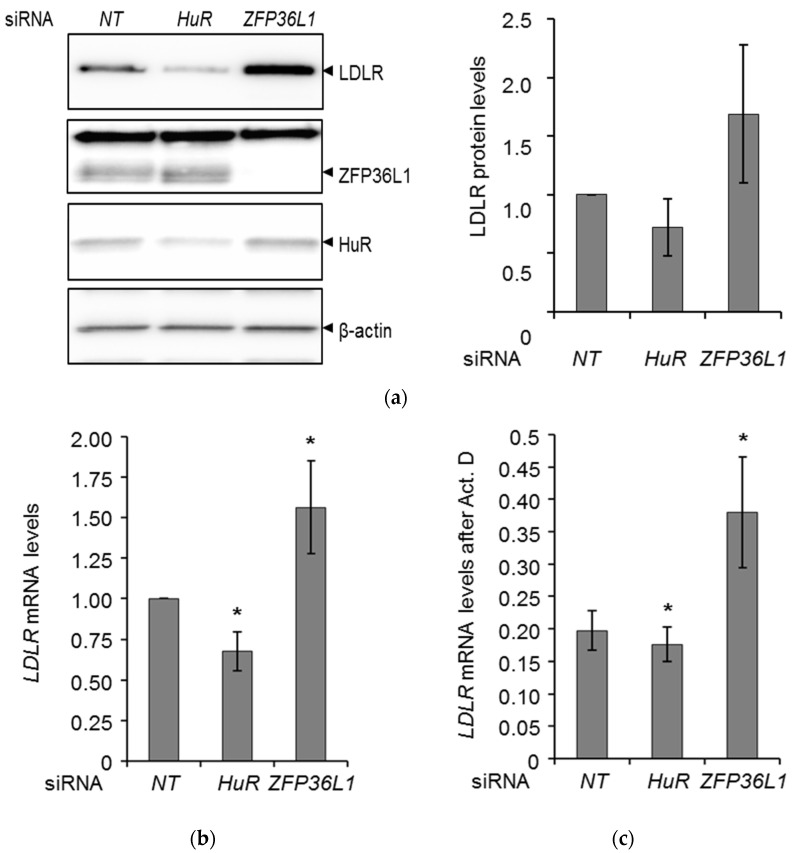
HuR and ZFP36L1 modulate endogenous low-density lipoprotein receptor (LDLR) expression and mRNA stability in HepG2 cells. (**a**) HepG2 cells were transfected with either a non-targeting control (*NT*), HuR or ZFP36L1 siRNAs. Twenty-four hours post transfection, cells were harvested and lysates subjected to immunoblotting using the indicated antibodies. One representative Western blot is shown (*n*  =  4). The graph in the right panel depicts the intensity of LDLR after normalization to the β-actin protein (*n* = 4). (**b**) Twenty-four hours post transfection, cells were harvested and total mRNA was extracted and subjected to quantitative RT-PCR (qPCR) using primers specific to LDLR, ZFP36L1, HuR, and GAPDH mRNA. Error bars show standard deviation of the mean. * *p* <  0.05, *n* = 9. (**c**) Twenty-four hours post transfection, cells were incubated with Act D, or not, for four hours. Cells were harvested at time zero (without Act D) and after four hours, respectively, and subjected to total RNA-extraction and qPCR of LDLR mRNA, normalized to GAPDH mRNA. The graph shows the LDLR mRNA level remaining after four hours Act D treatment, relative to *NT* siRNA-transfected control cells. Error bars show standard deviation of the mean. * *p*  <  0.05, *n* = 5.

HepG2 cells were transfected with HuR or ZFP36L1 siRNA and analyzed for HuR and ZFP36L1 protein levels in order to examine the knockdown efficiency of their respective siRNAs. HuR siRNA reduced HuR protein levels by only approximately 70% (Figure 1a). In contrast, siRNA directed against ZFP36L1 efficiently reduced ZFP36L1 protein level (Figure 1a). Next, the impact of the reduction in HuR and ZFP36L1 protein level on LDLR expression was examined. Figure 1a and Figure S1 show that a siRNA-mediated decrease in HuR levels reduced LDLR protein and its mRNA approximately 30% (Figure 1b) though its mRNA stability was only slightly decreased (Figure 1c). The depletion of ZFP36L1 enhanced LDLR protein (Figure 1a and Figure S1) and mRNA expression twofold (Figure 1b) and were echoed by increased mRNA stability (Figure 1c).

### 2.2. HuR and ZFP36L1 Contribute to TCN-Mediated Stabilization of LDLR mRNA

**Figure 2:**
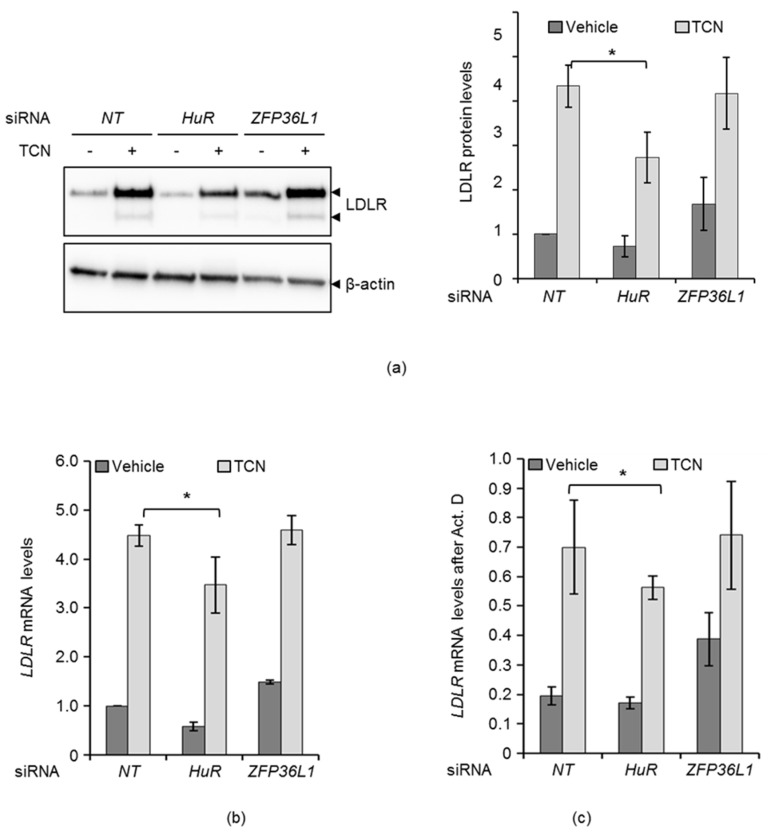
HuR and ZFP36L1 contribute to triciribine (TCN)-mediated stabilization of LDLR mRNA. (**a**) HepG2 cells were transfected with either a non-targeting *(NT),* 1HuR or ZFP36L siRNA. Twenty-four hours post transfection, cells were treated with or without TCN and harvested after four hours. Lysates were subjected to immunoblotting with the indicated antibodies. One representative blot is shown (*n*  =  4). The graph in the right panel depicts the intensity of LDLR after normalization to the β-actin protein (*n* = 4). (**b**) A similar experiment, as described in (**a**), was performed and cells were subjected to total RNA extraction and qPCR of LDLR mRNA, normalized to GAPDH. Error bars show standard deviation of the mean. * *p*  <  0.05, *n* = 3. (**c**) Twenty four hours post transfection, cells were added TCN, or not, for four hours before being exposed to Act D. Cells were harvested at time zero (without Act D) and after four hours with Act D, respectively, and subjected to total RNA-extraction and qPCR of LDLR mRNA, normalized to GAPDH mRNA. The graph shows the LDLR mRNA level remaining after four hours Act D treatment in vehicle and TCN-treated cells. Error bars show standard deviation of the mean. * *p*  <  0.05.

The impact of HuR- and ZFP36L1-specific siRNAs on the ability of TCN to stabilize LDLR mRNA were examined to explore the potential roles of HuR and ZFP36L1 in modulating the TCN-induced LDLR mRNA stability. HuR depleted cells exhibited reduced LDLR protein and mRNA expression in response to TCN, as shown in Figure 2a,b and Figure S1. Consistently, TCN increased the turnover rate of LDLR mRNA in HuR-depleted cells (Figure 2c). These data indicate a role for HuR as a mediator of the stabilizing effect of TCN on LDLR mRNA.

In ZFP36L1-depleted cells, TCN had no effect on LDLR protein or mRNA (Figure 2a,b), or its mRNA turnover (Figure 2c), contrary to HuR. These results imply that TCN inhibits the LDLR mRNA-degrading activity of ZFP36L1, an event that would contribute to the stabilization of LDLR mRNA by TCN.

### 2.3. TCN Induces Phosphorylation of ZFP36L1 Downstream of ERK

**Figure 3:**
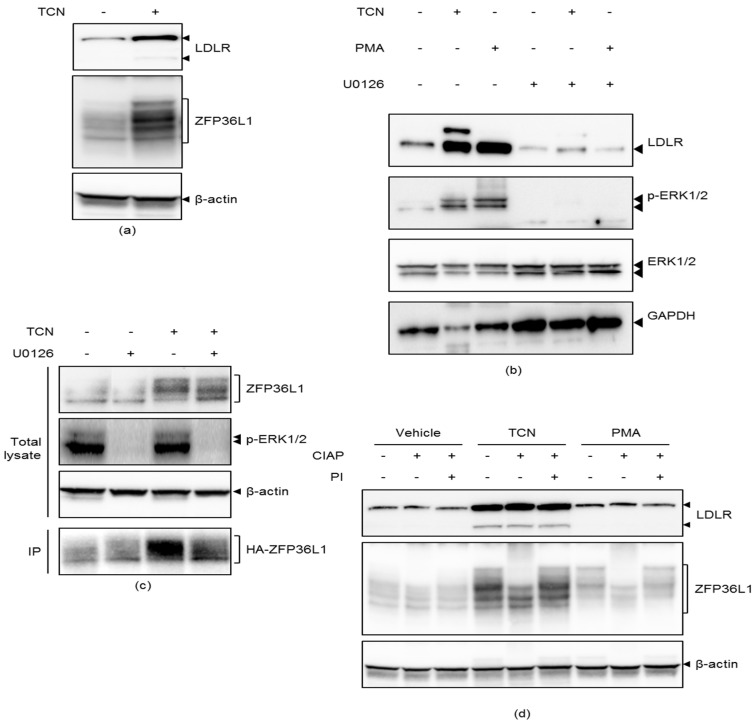
TCN induces phosphorylation of ZFP36L1 downstream of ERK. (**a**) HepG2 cells were exposed to TCN for four hours and lysates were immunoblotted with the indicated antibodies. One representative blot is shown, (*n* = 3). (**b**) Cells were exposed to U0126 for one hour before treatment with TCN or phorbol 12-myristate 13-acetate (PMA) for four hours. Lysates were immunoblotted with the indicated antibodies. (**c**) Cells were transfected with pcDNA4/TO-HA-ZFP36L1 and the day after transfection added U0126 for one hour, prior to addition of TCN for four hours. Lysate were subjected to immunoblotting with the indicated antibodies. IP: immunoprecipitation. (**d**) Cells were exposed to TCN for four hours or PMA for one hour. 25 µg of total lysate were treated as indicated and subjected to immunoblotting using the indicated antibodies. *CIAP* (calf intestinal alkaline phosphatase), *PI* (phosphatase inhibitor). One representative blot is shown, (*n* = 3).

TCN-treated cells were examined for the gel mobility behavior of ZFP36L1 as a proxy for its phosphorylation status and, thus, its activity in order to reveal whether TCN phosphorylates ZFP36L1. TCN treatment led to a prominent ZFP36L1 gel mobility shift, an event that was associated with an increased LDLR protein expression, as shown in Figure 3a. If the gel mobility shift of ZFP36L1 is due to ERK dependent phosphorylation it should be lost upon treatment with the MEK/ERK inhibitor U0126. Indeed, upon the co-treatment of cells with U0126, TCN was unable to induce ZFP36L1 phosphorylation (Figure 3b,c). Consistent results on ZFP36L1 gel mobility shift were obtained in HA-ZFP36L1 transfected cells, both in total lysate and HA-ZFP36L1 immunoprecipitated samples (Figure 3c). The cell extracts of PMA and TCN-treated HepG2 cells were incubated with calf intestinal alkaline phosphatase (CIAP), in the presence or absence of phosphatase inhibitors (PI) prior to analysis on SDS-PAGE, to ascertain that the observed TCN-induced changes in the gel mobility of ZFP36L1 occurs as a result of its phosphorylation (Figure 3d). Cell extracts from PMA-treated cells were included in this assay because PMA has been shown to induce phosphorylation-induced gel mobility retardation of ZFP36L1 [15]. CIAP eliminated the TCN-induced gel mobility retardation of ZFP36L1, an event that was inhibited when cell extracts were co-treated with PI, as can be seen in Figure 3d. Thus, TCN induces phosphorylation and a gel mobility shift of ZFP36L1.

### 2.4. TCN-Induced ZFP36L1 Phosphorylation is Mediated by the ERK Downstream Target RSK1

**Figure 4:**
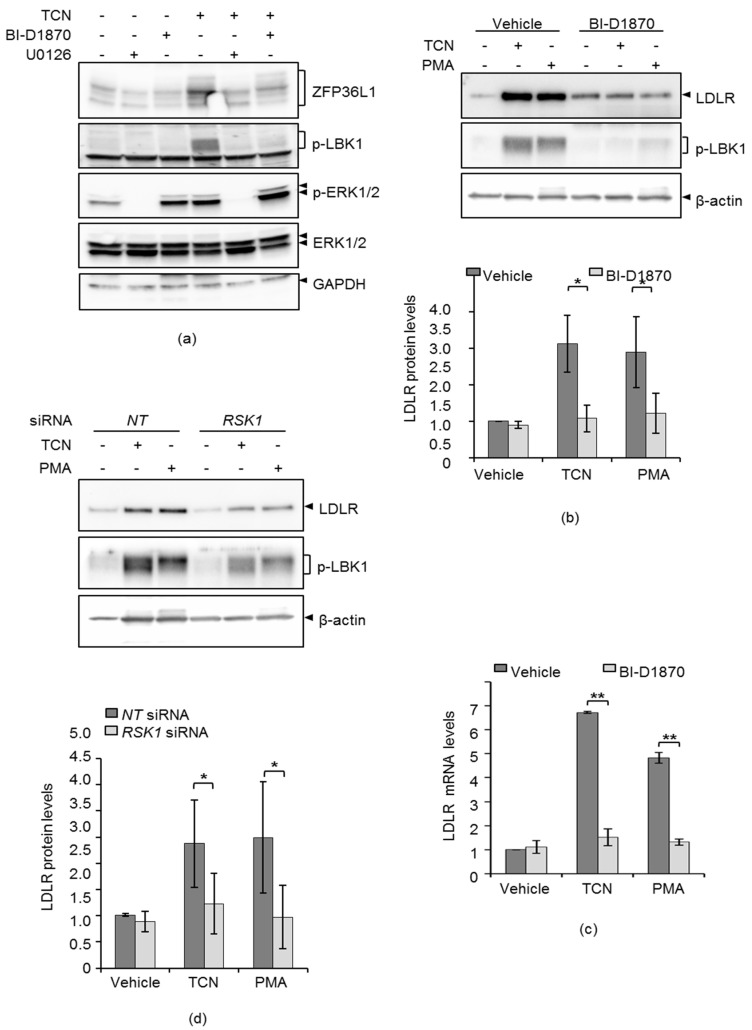
TCN-induced ZFP36L1 phosphorylation is mediated by the ERK downstream target RSK1. (**a**) HepG2 cells were treated with either U0126, BI-D1870 or not for one hour prior to the addition of TCN for four hours. Lysates were immunoblotted with the indicated antibodies. (**b**) HepG2 cells were treated with BI-D1870 or not for one hour prior to addition of TCN for four hours or PMA for one hour. PMA was used as a positive control in this assay. Lysates were subjected to immunoblotting with the indicated antibodies. The graph below the blot depicts the intensity of LDLR after normalization to the β-actin protein, * *p* < 0.05, *n* = 3. (**c**) HepG2 cells from a duplicate experiment, as described in (**b**) were harvested and subjected to total RNA-extraction and qPCR of LDLR and GAPDH mRNA, ** *p* < 0.005, *n* = 3. (**d**) HepG2 cells were transfected with either a non-targeting control (*NT*) or RSK1 siRNAs. Twenty-four hours post transfection, TCN or PMA were added for four hours and lysate were subjected to immunoblotting using the indicated antibodies. The graph below the blot depicts the intensity of LDLR after normalization to the β-actin protein (*n* = 3). Error bars represent standard deviation from the mean. * *p* <  0.05, *n* = 3.

The possibility that TCN-induced phosphorylation of ZFP36L1, an event that is dependent on ERK, could be mediated by RSK1, was considered [15]. To examine this assumption, HepG2 cells were treated with TCN in the absence or presence BI-D1870, an inhibitor of RSK1, and then analyzed for phosphorylation of ZFP36L1. As shown in Figure 4a, TCN induced ZFP36L1 phosphorylation and increased the activity of RSK1, as judged by the phosphorylation of its substrate LKB1. Indeed, co-treatment of cells with either U0126 or BI-D1870 reduced ZFP36L1 phosphorylation and abolished RSK1-activity. Consistently, cells that were treated with TCN or PMA, had increased LKB1 phosphorylation, as well as LDLR protein expression, an event that was abrogated in BI-D1870 treated cells (Figure 4b and Figure S2). The LDLR mRNA level consistently echoed that of its protein (Figure 4c). In order to confirm the results using RSK1-inhibitor BI-D1870 presented in Figure 4b), siRNA targeting RSK1 was performed and the abrogated effects of TCN and PMA on LDLR protein levels were also seen in RSK1-depleted cells (Figure 4d and Figure S3). These results show that TCN requires the activity of RSK1 to induce ZFP36L1 phosphorylation, an event that is associated with TCN-induced LDLR mRNA.

### 2.5. TCN Inhibits Association of ZFP36L1 with CNOT7

**Figure 5:**
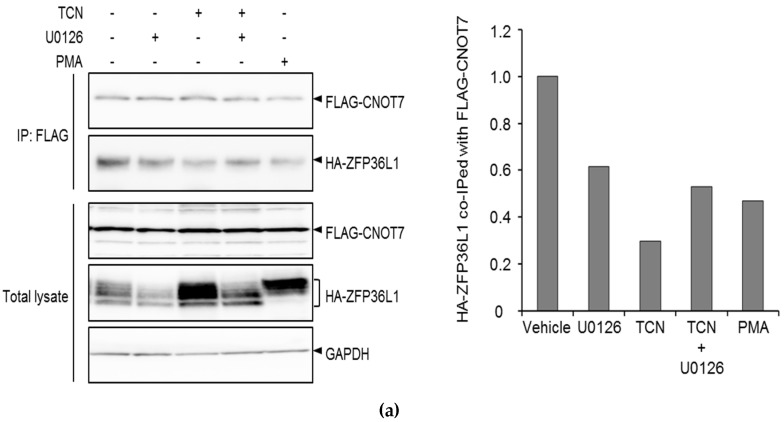

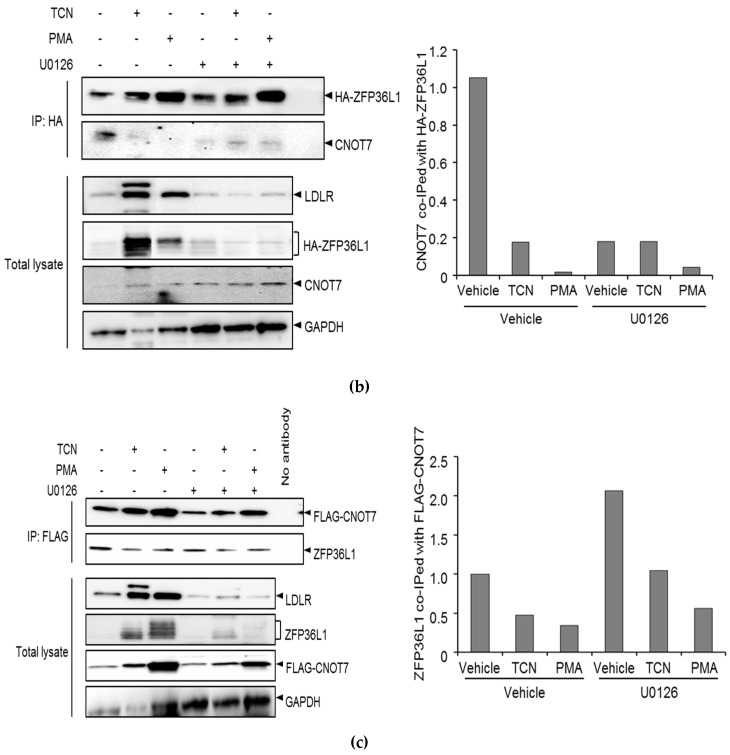
TCN inhibits association of ZFP36L1 with CNOT7. (**a**) HepG2 cells were co-transfected with FLAG-CNOT7 and HA-ZFP36L1. (**b**) HepG2 cells were transfected with HA-ZFP36L1. (**c**) HepG2 cells were transfected with FLAG-CNOT7. (**a**–**c**). One day post transfection cells were treated with or without U0126 for one hour before exposure to TCN or PMA for four hours. Lysate were subjected to immunoprecipitation with the indicated antibodies and retrieved immunocomplexes and total lysate were subjected to immunoblotting with the indicated antibodies. IP: immunoprecipitation.

Adachi et al. [15] demonstrated that the destabilizing effect of ZFP36L1 on LDLR mRNA requires the binding of the CCR4-NOT-deadenylase complex to its 3‘UTR. Upon phosphorylation of ZFP36L1, it dissociates from the CCR4-NOT complex and consequently stabilizes LDLR mRNA [15]. Therefore, three co-immunoprecipitation experiments were performed in order to examine whether TCN affected the interaction between ZFP36L1 and CNOT7. HepG2 cells were first co-transfected with FLAG-CNOT7 and HA-ZFP36L1 and then treated without, or with, TCN or PMA. Cell lysates were subsequently immunoprecipitated with anti-FLAG antibodies and the retrieved immunocomplexes were examined by blotting for HA-ZFP36L1 (Figure 5a). PMA treatment was used as the positive control for the modulation of CNOT7 and ZFP36L1 interaction [15]. TCN and PMA phosphorylates ZFP36L1, an event that is abolished upon co-treatment with U0126 (total lysate in Figure 5). As can be seen in Figure 5, CNOT7 immunocomplexes contained ZFP36L1 indicating that these two proteins interact. Importantly, the exposure of cells to TCN or PMA reduced the level of ZFP36L1 in CNOT7 immunoprecipitates. Furthermore, co-treatment with U0126 partially reversed the inhibitory effect of TCN on CNOT7 and ZFP36L1 interaction (Figure 5a).

In Figure 5b, the cell lysates were transfected with HA-ZFP36L1 and immunoprecipitated with anti-HA antibodies and the retrieved immunocomplexes were examined by blotting for endogenous CNOT7. HA-ZFP36L1 immunocomplexes contained CNOT7 indicating interaction between these two proteins. The exposure of cells to TCN or PMA reduced the level of CNOT7 in ZFP36L1 immunoprecipitates. Additionally, co-treatment with U0126 increased the interaction between CNOT7 and ZFP36L1 when compared to TCN- and PMA-treated cells.

Figure 5c shows cell lysates that were transfected with FLAG-CNOT7 and immunoprecipitated with anti-FLAG antibodies and the retrieved immunocomplexes were examined by blotting for endogenous ZFP36L1. CNOT7 immunocomplexes contained ZFP36L1, indicating again that these two proteins interact. Importantly, the exposure of cells to TCN or PMA reduced the level of ZFP36L1 in CNOT7 immunoprecipitates. Furthermore, co-treatment with U0126 partially reversed the inhibitory effect of TCN on CNOT7 and ZFP36L1 interaction (Figure 5c). Together, these complementary data presented in Figure 5a-c show that the interaction between ZFP36L1 and CNOT7 is reduced upon TCN and PMA-treatment. Inhibiting ERK1/2 with the co-treatment of U0126 increases the binding affinity of ZFP36L1 and CNOT7, compared to TCN- and PMA-treatment alone (Figure 5a–c).

### 2.6. TCN Induces Translocation of Nuclear HuR to the Cytoplasm in an ERK-Dependent Manner

**Figure 6:**
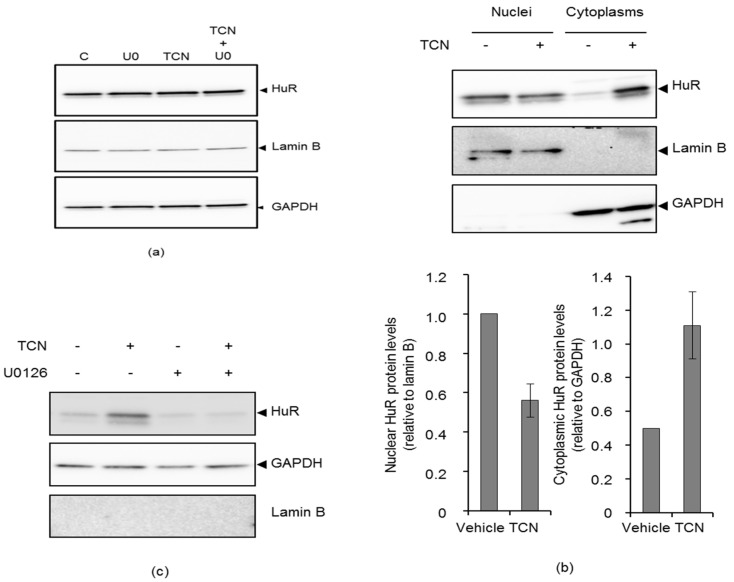

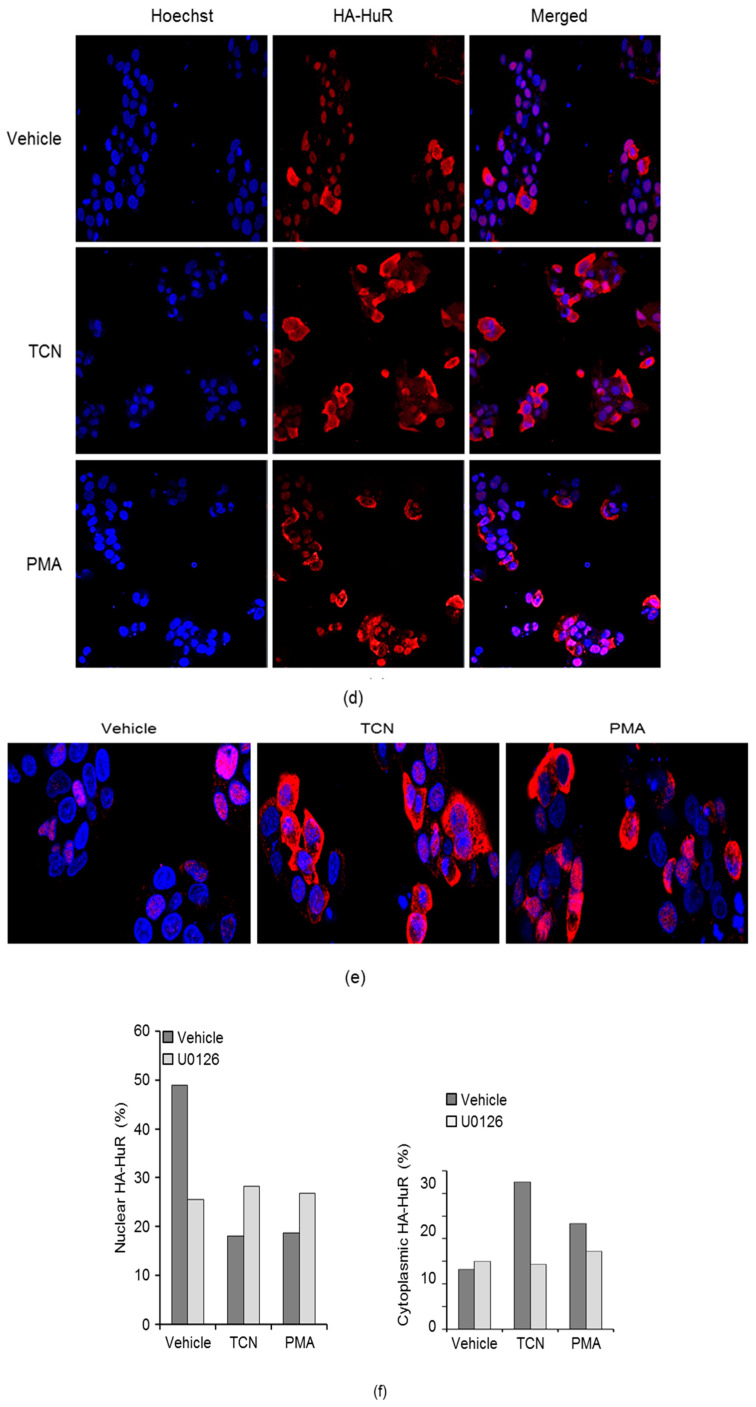
TCN induces translocation of nuclear HuR to cytoplasm in an ERK-dependent manner. (**a**) HepG2 cells were treated with, or without U0126 for one hour prior to addition of TCN or not for four hours and total lysate were immunoblotted with the indicated antibodies. (**b**) The cells were treated with TCN for four hours and subjected to subcellular fractionation with NE-PER™ Nuclear and Cytoplasmic Extraction Reagents. Nuclear and cytoplasmic fractions were immunoblotted with the indicated antibodies. (**c**) Cells were treated with, or without, U0126 for one hour prior to the addition of TCN for four hours and subjected to subcellular fractionation and the cytosolic fraction was immunoblotted with the indicated antibodies. (**d**) HA-HuR transfected cells were exposed to TCN or PMA for four hours and further examined by confocal immunofluorescence microscopy with anti-HA. (**e**) Merged picture of HA-HuR and nuclei (Hoechst) in cells treated as described in (**d**) and with an increased resolution compared to (**d**). Red: HA-HuR, Alexa fluor 647, blue: nuclei, Hoechst. One representative experiment is shown (*n* = 3). (**f**) Nuclear and cytoplasmic HA-HuR was localized in 600–900 cells for each treatment. The proportion of cells with nuclear and cytoplasmic HA-HuR, respectively, to the total number of cells counted, are shown (*n* = 1).

The effect of TCN on HuR localization was examined because HuR exerts its stabilizing effect on target mRNA after translocation to cytoplasm [23,24,27]. PMA treatment served as a positive control, since it has been shown to induce translocation of HuR from nucleus to cytoplasm [28]. Western blot analysis using whole-cell lysates revealed no obvious change in the total HuR expression upon TCN treatment (Figure 6a), but, upon subcellular fractionation, a two-fold increase in HuR protein levels was detected in the cytoplasm (Figure 6b), similar to that for PMA (Figure S4). Co-treatment of TCN-exposed cells with U0126 prominently reduced HuR abundance in the cytoplasm, as judged from the western blot presented in Figure 6c and Figure S5. The effect of TCN on the intracellular localization of transfected HA-HuR was also examined by immunofluorescence microscopy to confirm the fractionation data. In untreated cells, HuR localized mainly to nuclei, whereas upon TCN or PMA-exposure HuR localization was more prominent in the cytoplasm (Figure 6d–f). In order to obtain an overall picture of the cellular HuR localization upon TCN or PMA treatment, HA-HuR was examined in between 600 and 900 cells for the different treatments. The proportion of cells with cytoplasmic HuR-staining prominently increased upon TCN and PMA-treatment, when compared to untreated cells (Figure 6f). Nuclear HuR was accordingly reduced upon TCN and PMA exposure (Figure 6f). In addition, TCN and PMA-treated cells co-treated with U0126 had an equal proportion of HuR in nuclei and cytoplasm for all treatments (Figure 6f). In sum, these results show that TCN promotes the nuclear export of HuR to cytoplasm in an ERK-dependent manner.

## 3. Discussion

Pharmacological approaches that increase LDLR expression are required in order to treat hypercholesterolemia and orally ingested small molecule therapy such as statin therapy is the most convenient approach. Small molecule-induced stabilization of LDLR mRNA represents a very attractive approach to increase LDLR protein expression in order to treat hypercholesterolemia. Previous studies reported that the LDLR mRNA is labile, but that it can be stabilized in the presence of different small molecule compounds, such as berberine [5], canadine [11], AICAR [14], gemfibrozil [29], chenodeoxycholic acid [8] and triciribine [12]. While the mechanism of LDLR mRNA stabilization by canadine and gemfibrozil is still unknown, triciribine (TCN) shares similarities to berberine, AICAR, and chenodeoxycholic acid by stabilizing LDLR mRNA through an ERK1/2-dependent pathway, and involve the LDLR 3′UTR [5,8,12,14].

In the present study, a targeted approach was utilized to examine the role of HuR and ZFP36L1 in TCN-induced LDLR mRNA stabilization. These two proteins were considered to be likely candidates, because (1) they have been reported to modulate LDLR mRNA stability in response to agents that activate ERK and (2) the mechanisms underlying their regulatory effect on *LDLR* mRNA stability have been partially resolved [9,13,14,15]. To explore the potential roles of HuR and ZFP36L1 in modulating the TCN-induced LDLR mRNA stability, the impact of HuR- and ZFP36L1-specific siRNAs on the ability of TCN to stabilize LDLR mRNA were examined. Although HuR has been identified as a stabilizing protein for LDLR mRNA [9,13,14], Li et al., 2009, reported no modulation of LDLR mRNA level upon its depletion in HepG2 [13]. At contrary, Yashiro et al. reported that HuR depleted HepG2 cells showed a prominent decrease in LDLR mRNA and a slightly less stable mRNA [14]. In this report the knockdown efficiency of HuR was 70% and the effect on LDLR mRNA stability upon HuR-depletion was less prominent than that reported by Yashiro et al. [14]. Differences between these studies could be due to the different efficiency of HuR knockdown as the protein is ubiquitously expressed and apparently stable [30]. However, TCN prominently reduced the expression and stability of LDLR mRNA upon HuR-depletion. These data indicate a role for HuR as a mediator of the stabilizing effect of TCN on LDLR mRNA. Although predominantly nuclear, HuR shuttles between the nucleus and the cytoplasm. Cytoplasmic HuR have been reported to mediate the stabilization and increased translation of bound mRNA upon stimulation by agents that induce its translocation from nuclei to cytoplasm [27]. Interestingly, TCN prominently increased the level of HuR in the cytoplasm, whereas co-treatment of the cells with the ERK-inhibitor U0126 reversed this effect, as judged from immunoblotting (Figure 6b–c and Figures S4 and S5). Confocal immunofluorescence data confirmed the biochemical fractionation data and strongly suggest that HuR translocate from the nucleus to the cytoplasm upon TCN treatment in an ERK-dependent manner and protects LDLR mRNA from the mRNA decay machinery (Figure 6d–f).

For the study of ZFP36L, attention was focused on ZFP36L1 isoform, because, although both isoforms have been shown to control the stability of LDLR mRNA via the ERK pathway, only the ERK-dependent phosphorylation of endogenous ZFP36L1 has been rigorously examined [15]. In this report, it was shown that depletion of ZFP36L1 enhanced LDLR protein (Figure 1a and Figure S1) and mRNA expression twofold which was echoed by an increase in mRNA stability (Figure 1b,c) and confirms ZFP36L1 as a LDLR mRNA-destabilizing protein in HepG2 cells [9,13,14,15]. ZFP36L1-depleted cells had however a similar increase in LDLR mRNA stability as compared to control cells upon TCN-treatment (Figure 2). These observations could be explained by two equally plausible scenarios: (A) ZFP36L1 is not involved in mediating the stabilizing effect of TCN on LDLR mRNA. If this is the case, ZFP36L1-depleted cells treated with TCN should have increased LDLR mRNA stability as compared to *NT* control cells, according to that seen in untreated ZFP36L1-depleted cells or (B) TCN inhibits the LDLR mRNA-degrading activity of ZFP36L1, an event that would contribute to the stabilization of LDLR mRNA by TCN. If this is the case, then it is expected, as we show, that ZFP36L1 depletion would not affect TCN-induced LDLR mRNA stability as inactivation of ZFP36L1 by TCN precedes its knockdown by siRNA.

PMA has been shown to partially increase LDLR-mRNA stability through ERK-induced ZFP36L1 phosphorylation [15]. To further reveal the molecular mechanisms underlying ZFP36L1-induced stabilization of LDLR mRNA by TCN, I took the advantage of utilizing PMA as a positive control. To this end, TCN-treated cells were examined for the gel mobility behavior of ZFP36L1 as a proxy for its phosphorylation status. Indeed, it was shown that TCN induced a gel mobility shift of ZFP36L1 due to phosphorylated forms of the protein which disappeared upon inhibiting ERK (Figure 3). It has been shown that ERK induces phosphorylation of ZFP36L1 through its downstream target, p90 ribosomal S6 kinase 1 (RSK1) [15]. Consistently, it was demonstrated that TCN-induced phosphorylation of ZFP36L1 is dependent on RSK1 activity (Figure 4 and Figures S2 and S3). These results, together with present data showing that TCN-mediated stabilization of LDLR mRNA, require ERK and its downstream target RSK1, place ERK as the common denominator linking TCN to regulation of ZFP36L1 phosphorylation. ZFP36L1 has been shown to recruit the CCR4-NOT deadenylase complex to LDLR 3′ UTR through interaction with CNOT7, which results in its mRNA degradation [15]. Adachi et al. demonstrated that PMA increased LDLR mRNA stability through the phosphorylation of ZFP36L1 and its subsequent release from CNOT7 [15]. Therefore, three complementary co-immunoprecipitation experiments were performed in order to examine whether TCN could modulate the interaction between ZFP36L1 and CNOT7. Indeed, cells exposed to TCN had reduced interaction of ZFP36L1 and CNOT7, as shown by co-immunoprecipitation experiments presented in Figure 5a–c. Furthermore, the inhibition of ERK with U0126 partially reversed the inhibitory effect of TCN on CNOT7 and ZFP36L1 interaction (Figure 5a–c). Taken together, these results indicate that TCN exerts an inhibitory effect on the association of ZFP36L1 with CNOT7 in an ERK-dependent manner.

In conclusion, this report shows that the stabilizing effect of TCN is partially mediated by the activation of HuR and inactivation of ZFP36L1. Approximately 30% of the TCN induction on LDLR mRNA stability could be explained by these two proteins. An even more prominent effect of HuR-depletion would be expected if the knockdown effect was more efficient. Other transacting proteins are likely involved in the TCN-induced stabilization of LDLR mRNA and the complete picture could only be elucidated through a more comprehensive study where screening of proteins that binds to LDLR 3‘UTR upon TCN treatment are identified and functionally analyzed with regards to their ability to modulate LDLR mRNA stability.

## 4. Materials and Methods

The cells were treated with chemicals at the final concentration of 1 µM Triciribine (TCN) (Selleckchem, Houston, TZ, USA), 100 ng/mL 12-O-Tetradecanoylphorbol-13-acetate (PMA) (Sigma-Aldrich, Saint Louis, MO, USA), 10 µM RSK Inhibitor BI-D1870 (Selleck Chemicals LLC, Saint Louis, MO, USA), 10 µM ERK-inhibitor U0126, and 5 ug/mL Actinomycin D (Act D). Paraformaldehyde, saponin, and sodium orthovanadate (Na_3_VO_4_) were all from Sigma–Aldrich (St. Louis, MO, USA). Hoechst 33342 was from Invitrogen (ThermoFisher Scientific, MA, USA).

Antibodies against LDLR (#3839) (BioVision, Milpitas, CA, USA), β-actin (ab8227) (Abcam, Cambridge, UK) Phospho-LKB1/STK11 (S428)(AF5636) (R&D Systems, Minneapolis, MN, USA), GAPDH (G9295) Sigma-Aldrich, Saint Louis, MO, USA), HuR (ELAVL1)(sc-5261) and Lamin B (sc-6217) (Santa Cruz Biotechnology, Dallas, TX, USA), ERK1/2 (#9102), pERK (#4370) and ZFP36L1/L2 (BRF1/2) (#2119) (Cell Signaling, Beverly, MA, USA), anti-cnot7 (H00029883-A01), (Abnova, Taipei, Taiwan), anti-FLAG (F7425) and anti-HA (#71–5500) (Thermo Fisher Scientific, Waltham, MA, USA).For confocal immunoflouroscence microscopy primary anti-HA (1:100) (#71–5500), with secondary Alexa Fluor 647-conjugated anti-rabbit (715–546–150 Jackson ImmunoResearch, West Grove, PA, USA) (1:200), were used, respectively.

### 4.1. Cell Culture and Treatments

HepG2 cells (European Collection of Cell Cultures, Salisbury, UK) were cultured on collagen-coated culture vessel (BD Biosciences, San Jose, CA, USA) in HyClone Minimum Essential Medium (GE Healthcare Life Sciences, Pittsburg, PA, USA) supplemented with 10% fetal bovine serum (FBS) and 2 mM L-glutamine (Sigma-Aldrich, Saint Louis, MO, USA), 50  U/mL penicillin and 50  µg/mL streptomycin (GE Healthcare Life Sciences, Pittsburg, PA, USA ), and non-essential amino acids (Biowest, Nuaillé, France). All of the cells were cultured at 37 °C in an atmosphere with 5% CO_2_ and 95% humidity. All drugs were dissolved in DMSO and added to cell cultures, such that the final concentration of DMSO was kept at 0.1% (*v/v*). Control cultures were treated with DMSO alone at 0.1% (*v/v*).

### 4.2. DNA Constructs

pcDNA-HA-HuR (ELAVL1), pcDNA4-HA-ZFP, and pcDNA4-FLAG-cnot7 were amplified from HepG2 derived cDNA with the following primers containing restriction-sites marked in bold and cursive. The plasmid constructs were cloned using pcDNA™4/TO Mammalian Expression Vector (Invitrogen) (Table 1).

### 4.3. Short-Interfering Oligonucleotides (siRNA)

AllStars negative human siRNA or human ZFP36L1_5 (SI04194204), human ELAVL1_1 (SI00300139) (Qiagen), or RPS6KA1 (RSK1) (SI02223060) using Lipofectamine RNAiMAX (Thermo Fisher Scientific) to cells at 70% confluency.

### 4.4. Transfections

The HepG2 cells were transfected with 312 ng DNA/cm^2^ cells at a 4.5:1 FuGENE HD transfection reagent: DNA ratio according to the manufacturer’s instructions (Promega, Madison, WI, USA). Transfection with empty vector served as control. Cells were treated with drugs at 16–24 h after transfection. For immunoprecipitation assay, 100 mm dishes containing 12 mL warm FBS were transfected with 4.6 ug HA-ZFP or FLAG-cnot7, with 42 µL FuGene HD. For gene knockdown studies, the HepG2 cells were transfected with 40 pmol gene-specific/10 cm^2^ well and 12 ul RNAiMaX.

### 4.5. Western Blot Analysis

Upon harvesting medium was removed and cells washed in 1xPBS, and either harvested directly in lysis buffer or after freezing to −20 °C. After protein concentration determination using Pierce BCA protein assay (Thermo Fischer Scientific, Waltham, MA, USA), equal amounts of proteins were resolved on a 4–20% SDS-PAGE, transferred to PDVF membrane (Bio-Rad, Hercules, CA, USA) and then subjected to immunoblotting. After densitometric scanning of the immunoblots, the band intensity of the protein of interest was normalized to that of GAPDH or β-tubulin and plotted relative to the control value (vehicle-treated cells).

### 4.6. Phosphorylation Assay

The cells were harvested by trypzination, resuspended in cold medium and sentrifuged for 6 min at 1250× *g*. Pellets were resuspended in 100 µL cold reaction buffer 1xAP buffer (50 mM Tris, pH 7.5, 1 mM MgCl2, 1% Triton X-100, Protease inhibitors), incubated on ice for 30 min, vortexed at 1500 RPM each tenth minutes, centrifuged at 15,000× *g* for 30 min at 4 °C. The supernatants were transferred to new tubes and the protein concentration was determined with BCA. 25 µg of lysates were treated with either 1xAP control buffer alone (C), 4.2 U CIAP in 1x Rx buffer (CIAP), or 25 U CIAP, 0.5 M NaF, 0.5 M beta-glycerophophate, 1 M Na_3_VO_4_ and 0.5 M EDTA (CIAP + PI) in a total volume of 20 μL. The samples were incubated 60 min at 37 °C, and then added SDS-loading buffer, denaturated, fractionated on 10% SDS-PAGE gel, and immunoblotted with the indicated antibodies.

### 4.7. Immunoprecipitation (IP)

The day after transfection, HepG2 cells were trypzinated and harvested in 800 uL co-IP buffer (1M Tris, pH 7.5, 2.5 M NaCl, 1M MgCl2, 10% NP40, 0.5 M NaF, beta-glycerophosphate, and protease inhibitors). The lysate was passed 15x through a 21-G needle, incubated on ice for 20–30 min, centrifuged for 30 min at 4 °C, supernatants were transferred to new tubes and protein concentration measured. For total lysate, 25 µg proteins were analyzed on a 4–20% SDS-PAGE. For IP assay, 600 µg lysate was brought to 800 µL with co-IP buffer and 10 µg anti-FLAG (F7425) and incubated with rotation over night at 4 °C. 80 μL Protein G Mag Sepharose (#28951379, GE) were incubated with the FLAG treated lysates for 90 min at 4 °C, washed with 3x co-IP buffer. Lysate-beads were incubated with 30 μL 1xSDS at 95 °C for 15 min. The denaturated lysate were withdrawn from the beads and fractionated on a 4–20% SDS-PAGE prior to immunoblotting.

### 4.8. Subcellular Fractionation

Biochemical cell fractionation of nuclei and cytoplasm was performed with NE-PER™ Nuclear and Cytoplasmic Extraction Reagents according to the manufacturers protocol (Thermo Fisher Scientific, Waltham, MA, USA).

### 4.9. Quantitative Real-Time PCR

The total RNA was isolated with the QIAamp RNA Isolation Kit (Qiagen, Hilden, Germany) and converted to cDNA using the AffinityScript QPCR cDNA Synthesis Kit (Agilent Technologies, Santa Clara, CA, USA). The cDNA was then used as the template for quantitative real-time polymerase chain reaction (qPCR) using Brilliant III Ultra-Fast QPCR Master Mix on Mx3005P QPCR system (Agilent technologies, Santa Clara, CA, USA) and the following PrimeTime Predesigned qPCR Assays (Integrated DNA Technologies, Coralville, IA, USA): human LDLR (Hs.PT.58.14599757), human GAPDH (Hs.PT.39a.22214836), human ZFP36L1 (Hs.PT.56a.20479742), human ELAVL1 (Hs.PT.58.22593583), and human RPS6KA1 (RSK1) (hsPT58.1350004). qPCRs were run in duplicate and GAPDH was used as the normalizing gene. The 2^−ΔΔCt^ method was used to calculate relative mRNA levels.

### 4.10. Analysis of HuR Expression by Confocal Immunofluorescence Microscopy

The samples were fixed in 10% paraformaldehyde followed by permeabilization using 0.1% Saponin. pcDNA4/TO-HA-HuR transfected cells were then incubated with anti-HA (1:200) (#71–5500) (Thermo Fisher Scientific, Waltham, MA, USA) overnight. Alexa Fluor 647-conjugated anti-rabbit (1:200 dilution; 715–546–150), (Jackson Immuno Research, Cambridge, UK) was used for visualization. Hoechst 33342 (Invitrogen) Thermo Fisher Scientific, Waltham, MA, USA) was used to stain DNA. All of the pictures were taken with a Zeiss LSM 700 confocal system at the same magnitude (40×). Data in Figure 6f refer to the localization of HA-HuR in nuclei or cytoplasms that was counted in between 600 to 800 cells for the different treatments. The localization of HA-HuR in each cell, either nuclear or cytosolic, was determined.

### 4.11. Statistical Analysis

Experiments with inefficient knockdown efficiency of target gene (below 70%) or in which the expected induction of LDLR mRNA by TCN (2–4 fold) failed, were dismissed from further statistical analysis. The differences in gene expression between different treatments were calculated with the mean of each treatment by unpaired, two-tailed Student‘s *t*-test, and a *p*-value < 0.05 was considered to be statistically significant. For mRNA stability calculations the differences in gene expression between time zero and four hours after Act D was examined by paired, two-tailed Student’s *t*-test, and a *p* value of <0.05 was considered as statistically significant. Experiments where LDLR mRNA deviate more than 5% from the expected mRNA (0.2) level after Act D treatment in *NT* control cells, were dismissed from further statistical analysis. All of the experimental replicates refer to biological replicates.

## Figures and Tables

**Table 1:** Oligonucleotides used for PCR-amplification of DNA constructs.

Primer Name	Sequence	Incorporated Restriction Site
ELAVL1_5-EcoR	5- TACA***GAATTC***TACAATGTCTAATGGTTATGAAGACCAC -3	EcoRI
ELAVL1_3-Xho	5-TACT***CTCGAG***GCGAGTTATTTGTGGGACTTGTT-3	XhoI
ZFP36L1_5-Bam	5-TACA***GGATCC***tcGATGACCACCACCCTCGTG-3	BamHI
ZFP36L1_3-Xho	5-TACT***CTCGAG***TGGCTTAGTCATCTGAGATGGAAAG-3	XhoI
CNOT7_Bam *	5-TACA***GGATCC**T*CCCTTGTGCCCTCACTATG-3	BamHI
CNOT7_Xho *	5-TACT***CTCGAG***AGCTTTCCCCACTCTCTGTC-3	XhoI
CNOT7-del1040-1053-S	5′-GCGGTGGATCCTCCATGCCAGCGGCAAC-3′	
CNOT7-del1040-1053-AS	5′-GTTGCCGCTGGCATGGAGGATCCACCGC-3′	

* Puts CNOT7 out of frame. CNOT7-del1040-1053-S and CNOT7-del1040-1053-AS primers (below) were used to put CNOT7 in frame.

## Supplementary Materials

The following are available online at Figure S1: The effect of ZFP36L1 and HuR on LDLR protein expression in HepG2 cells with and without TCN, Figure S2: LDLR-induced protein expression by TCN is abrogated by the RSK1-inhibitor BI-D1870, Figure S3: LDLR-induced protein expression by TCN is abrogated in RSK1-siRNA treated HepG2 cells, Figure S4: TCN induces translocation of nuclear HuR to cytoplasm, Figure S5: TCN induces translocation of nuclear HuR to cytoplasm in an ERK-dependent manner.
