# Supplementary material for: Triciribine Engages ZFP36L1 and HuR to Stabilize LDLR mRNA
Source: Molecules. 2020 Oct 1;25(19):4505. doi: 10.3390/molecules25194505 (PMC7583736; doi:10.3390/molecules25194505)
Supplement: Supplementary file 1 [file molecules-25-04505-s001.pdf]

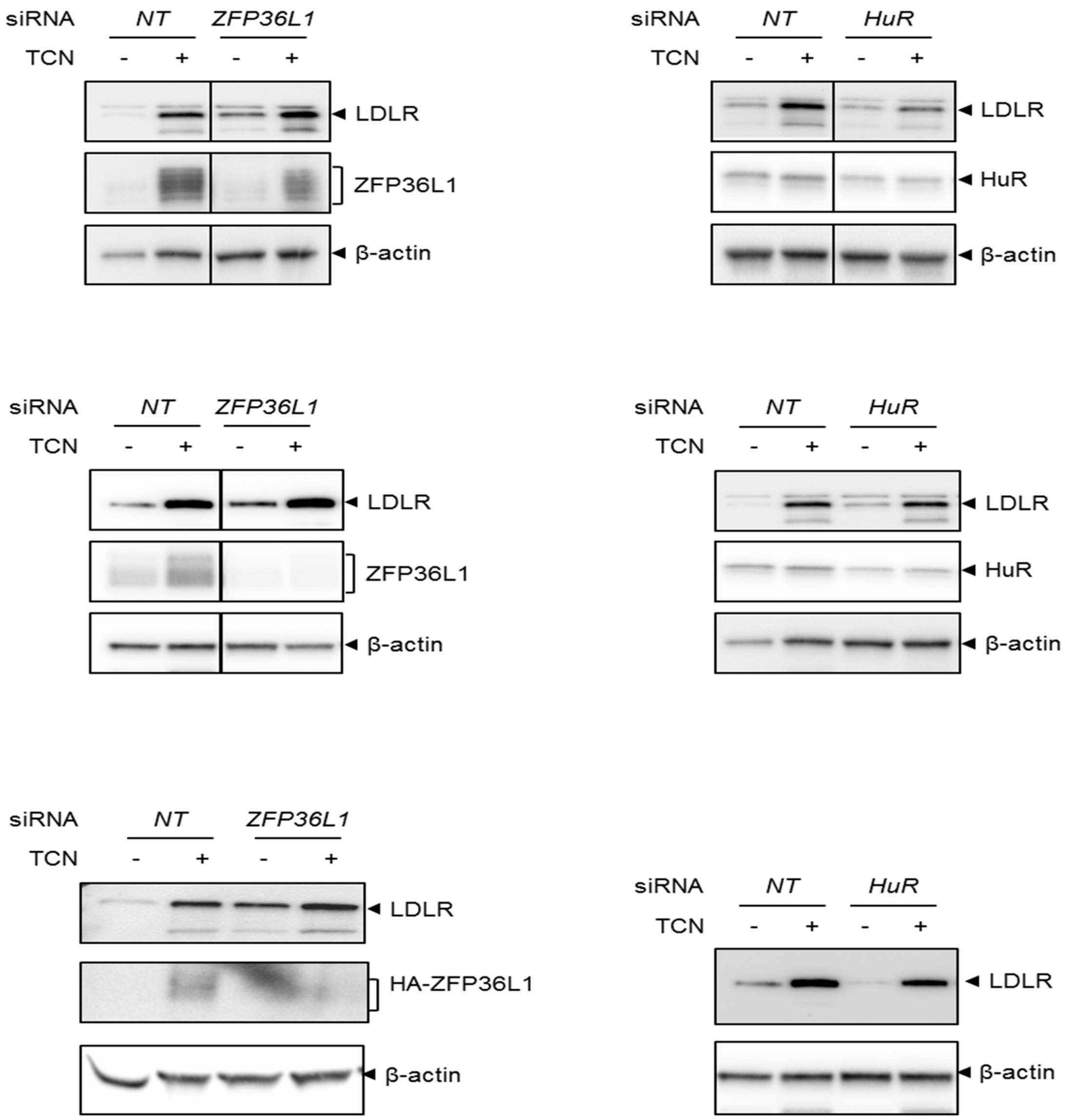

Figure S1. The effect of ZFP36L1 and HuR on LDLR protein expression in HepG2 cells with and without TCN. HepG2 cells were transfected with either a non-targeting *(NT), ZFP36L1* or *HuR* siRNA. Twenty four hours post transfection, cells were treated with or without TCN and harvested after four hours. Lysates were subjected to immunoblotting with the indicated antibodies. One representative blot is shown (n = 4).

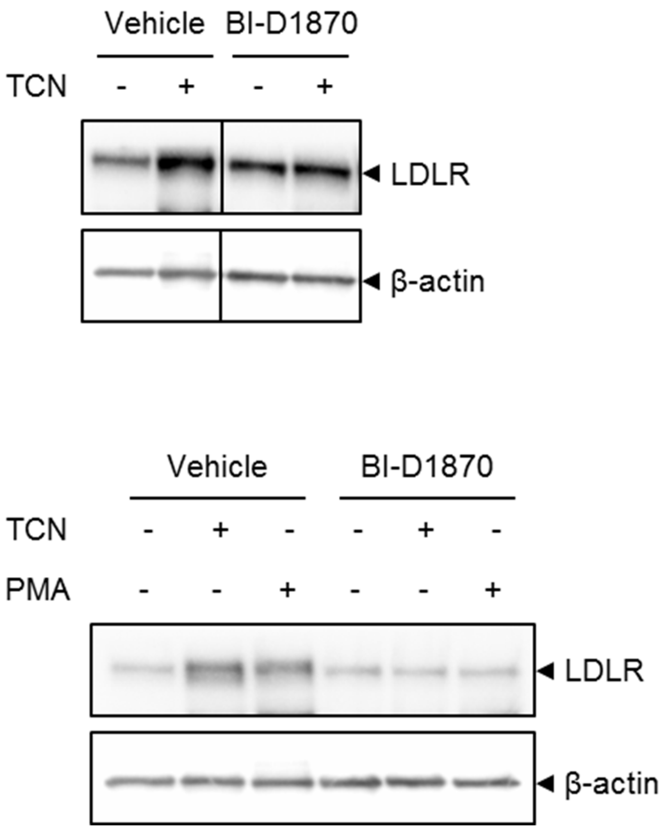

Figure S2. LDLR-induced protein expression by TCN is abrogated by the RSK1-inhibitor BI-D1870. HepG2 cells were treated with BI-D1870 or not for one hour prior to addition of TCN for four hours or PMA for one hour. PMA was used as a positive control in this assay. Lysates were subjected to immunoblotting with the indicated antibodies.

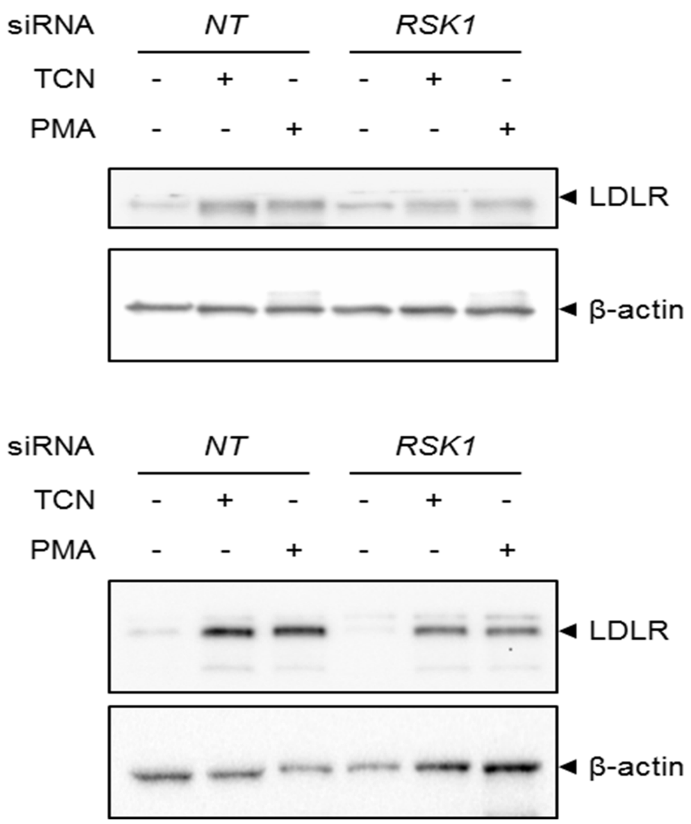

Figure S3. LDLR-induced protein expression by TCN is abrogated in RSK1-siRNA treated HepG2 cells. HepG2 cells were transfected with either a non-targeting control (*NT*) or RSK1 siRNAs. Twenty four hours post transfection, TCN or PMA were added for four hours and lysate were subjected to immunoblotting using the indicated antibodies.

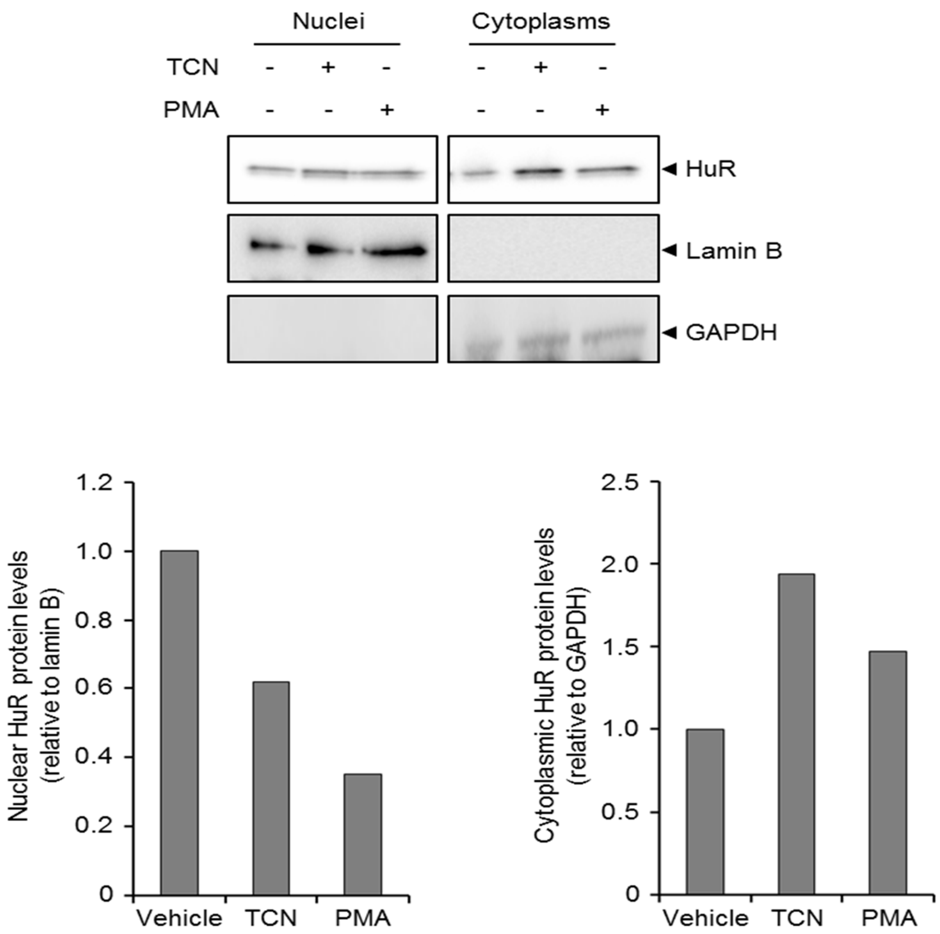

Figure S4. TCN induces translocation of nuclear HuR to cytoplasm.HepG2 cells were treated with TCN for four hours and subjected to subcellular fractionation with NE-PER™ Nuclear and Cytoplasmic Extraction Reagents. Nuclear and cytoplasmic fractions were immunoblotted with the indicated antibodies.

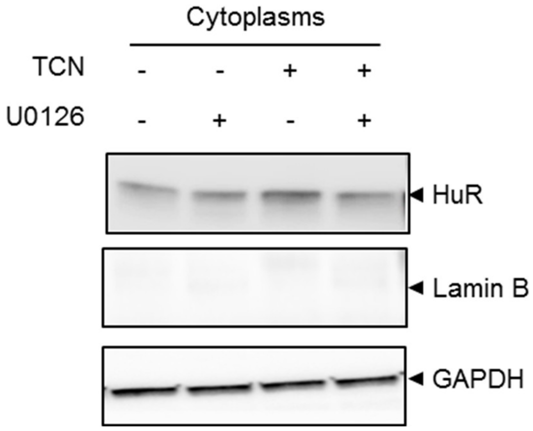

Figure S5. TCN induces translocation of nuclear HuR to cytoplasm in an ERK-dependent manner. Cells were treated with, or without, U0126 for one hour prior to addition of TCN for four hours and subjected to subcellular fractionation and the cytosolic fraction was immunoblotted with the indicated antibodies.
